# Enhancing external quantum efficiency in a sky-blue OLED by charge transfer via Si quantum dots

**DOI:** 10.1186/s11671-024-04171-w

**Published:** 2024-12-14

**Authors:** Zingway Pei, Han Yun Wei, Yi Chun Liu, Thiyagu Subramani, Naoki Fukata

**Affiliations:** 1 Graduate Institute of Optoelectronic Engineering, National Chung Hsing University, 145, Xingda Rd., Taichung, Taiwan (ROC); 2 International Center for Materials Nanoarchitectonics (MANA), National Institute for Materials Science (NIMS), 1-1 Namiki, Tsukuba, 305-0044 Japan; 3 Innovation and Development Center of Sustainable Agriculture (IDCSA), National Chung Hsing University, 145, Xingda Rd., Taichung, Taiwan (ROC)

**Keywords:** Quantum Dots, Quantum Efficiency, Phosphorescent, Light-Emitting Diode, Enhancement

## Abstract

Organic light-emitting diodes aim to achieve high efficiency by using excitons to achieve a 100% quantum efficiency (QE). However, developing functional organic materials for this purpose can be time-consuming. To address this challenge, a new method has been proposed to incorporate inorganic quantum dots into the organic luminescent layer to enable unlimited exciton formation and approach the 100% QE limit. Inorganic quantum dots are clusters of atoms that contain numerous thermally generated electrons and holes at conduction and valence bands. Immersed quantum dots act as charge generation centers, providing electrons and holes with unlimited amounts to form excitons. After radiative recombination, these excitons generate photons that cause internal QE to nearly 100%. This concept has been demonstrated using Silicon quantum dots (SiQDs) and phosphorescent materials. The average size of SiQDs is approximately 6 nm, and they are well-dispersed within the guest–host blue phosphorescent light-emitting materials. With only 5 × 10^–3^% (in weight) of SiQDs in the precursor, external QE increased from 2 to 17.7%, nearly a nine-fold enhancement. The prolonged decay time from 1.68 to 5.97 ns indicates that electrons are transferred from SiQDs to the luminescent materials. This universal method can be applied to green and red emissions with various inorganic quantum dots in different organic luminescent material systems.

## Introduction

Organic light-emitting diodes (OLED) are widely used in the display of smartwatches, cell phones, televisions, and laptop computer monitors due to OLED’s superior properties, such as being large-area, self-emissive, having a high contrast ratio, and being able to be made in flexible form [1]. In addition, OLED should be utilized in emerging areas such as augmented reality (AR), virtual reality (VR), indoor visible light communication, and bio-identifications [2, 3]. The brightness and efficiency of an OLED are crucial for various applications. They are closely linked to the radiative emission process. Electrons and holes are injected from the cathode and anode into the OLED and then transported to the luminescent material for radiative emission. The radiative emission recombines electrons and holes into photons. This process is evaluated by the quantum efficiency (QE), which indicates the percentage of electrons and holes transformed into photons, encompassing injection efficiency, transport efficiency, balance of electron and hole numbers, exciton formation efficiency, and radiative recombination efficiency of carriers [4].

The brightness depends on the number of emitted photons. The earliest fluorescent material emits photons by exciton recombination through the singlet state (S_1_) to ground state (S_0_) transition. The participation of S_1_ exciton is 25%, limiting the QE to be 25%. Another 75% of excitons belong to the triplet state (T_1_), which is not recombined radiatively due to the unpaired spin [5]. Several material systems are reported to achieve high efficiency and high brightness [6, 7], including but not limited to the guest–host phosphorescent materials [8], thermal activate delayed fluorescent (TADF), and type-II aligned luminescent materials for exciplex emissions [9, 10]. Phosphorescent materials are typically molecules with a transition metal at the center, facilitating metal–ligand charge transfer to ensure charge recombination through singlet and triplet states, thereby enhancing radiative recombination efficiency [11–15]. Theoretically, the allowance of both singlet and triplet transition in the phosphorescent material has the ultimate quantum efficiency (QE) of up to 100% [16–18]. The phosphorescent material is appropriately doped in a host material to prevent concentration quenching and form the guest–host luminescent system in a phosphorescent OLED [18, 19]. The TADF depends on a specially designed luminescent material or a material system that has a very small singlet and triplet states’ energy difference ($\Delta$E_S-T_). The excitons in the triplet states are transferred to singlet states through reverse inter-system crossing (RISC) by thermal energy [20–24]. By transferring the excitons in triplet states to singlet states, the theoretical QE is 100%.

To enhance the QE of an OLED, it is important to increase the number of charge carriers available for radiative recombination through device architecture, in addition to the complex material design and synthesis. One way to achieve this is by inserting a layer of indium-tin-oxide (ITO) or V_2_O_5_ between two luminescent layers to increase the number of charge carriers [25]. Electron–hole pairs generated within the V_2_O_5_ layer are separated and then injected into the corresponding luminescent layers by an applied voltage. Subsequently, they recombine radiatively with electrons or holes injected from the cathode or anode. Using an oxide semiconductor charge generation layer, the current efficiency increased from approximately 16–31 cd/A. With two V_2_O_5_ layers, the current efficiency increased to nearly 48 cd/A. By appropriately designing the emitting layer, considering material combinations, and replacing the V_2_O_5_ with MoO_3_, the QE can be improved to as high as 40% [26]. Besides the single-layer oxide semiconductor, various charge-generation layers have been reported. These include photovoltaic-type organic bulk heterojunction layers [27, 28] like CuPc–C60 or ZnPc–C60, an organic heterojunction donor–acceptor layer (HAT-CN: m-MTDATA) [29], MoO_3_–ZnO bilayers [30], organic–inorganic bilayers (C60/rubrene: MoO_3_) [31], and perovskite (CsPbBr_3_)-C60 bilayers [32]. The efficiency improved by these charge generation layers. It’s important to note that in some tandem devices, an increase in total thickness leads to a higher applied voltage. Along with the high operating voltage, the complexity of device fabrication also hinders the realization of CGLs. This work proposes and implements a device with a simple structure that leverages multiple CGLs to achieve high IQE without an increased operating voltage by using inorganic quantum dots.

We present a highly efficient blue organic light-emitting diode (OLED) that utilizes silicon quantum dots (SiQDs) as charge-generation centers (CGCs). This approach enables internal quantum efficiency (IQE) to be over 100%. The SiQDs, characterized by transmission electron microscopy (TEM), x-ray photoemission spectroscopy (XPS), and photoluminescence (PL) spectrum, have an average size of approximately 6 nm and are well-dispersed within the guest–host blue phosphorescent light-emitting materials. The XPS depth profile shows that the Si atoms are distributed throughout the layer. Moreover, the presence of SiO_2_ in the XPS indicates that the SiQDs have a core–shell structure, which allows them to be spatially separated within the luminous layer. While SiQDs exhibit red emission when excited by ultraviolet illumination, the blended SiQDs and blue phosphorescent exhibit sky-blue emission peaks at 436, 475, and 500 nm in the PL spectrum. To investigate the impact of SiQDs on OLED performance, we measured the electroluminescent spectrum on OLED devices with different SiQD concentrations. With only 5 × 10^–3^% (in weight) of SiQDs in the precursor, the QE increased from 2 to 17.7%. Notably, the operation voltage remained almost unchanged at this concentration, indicating that SiQDs do not affect the operation of an OLED. Time-resolved photoluminescence was used to investigate charge generation. We found that the decay time in the time-resolved PL (TR-PL) increased from 1.68 to 5.97 ns. By the charge-transition mechanism, the increased radiative decay time indicates the charge transfer from the SiQDs to the luminous material.

## Materials and methods

### Concept

**Fig. 1:**
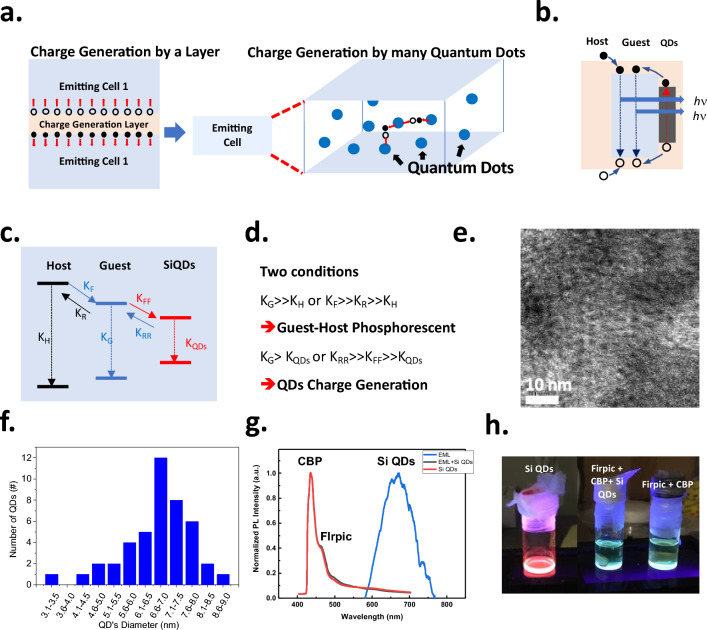
The proposed structure and physical properties of SiQDs. **a** The proposed system contains well-dispersed SiQDs as the unlimited CGCs. **b** The carrier donation path from SiQDs to the guest–host materials. **c** the expression of charge transfer inside a proposed system by transition rate. **d** The criteria for the transition rate between guest–host and SiQDs is to achieve high efficiency in the proposed SiQDs CGCs system. **e** The schematic expression and the TEM images of the SiQD. **f** The distribution of the diameter of the observed SiQDs. **g** The photoluminescence (PL) spectrum, and **h** the visible appearance of the SiQDs and SiQDs blended guest–host material system excited by UV light

The proposed structure, shown in Fig. 1a, exemplifies a significant deviation from the traditional approach. Rather than forming a charge generation layer (CGL), the Si quantum dots (SiQDs) are dispersed throughout the luminescent layer, allowing for electrons or holes provided by the SiQDs to form excitons at neighboring luminescent molecules (guest) and recombine radiatively to emit light. The SiQDs are covalent-bonded inorganic materials with a high density of states that contribute many carriers, as depicted in Fig. 1b. Ensuring the carriers in SiQDs transfer effectively to the luminescent molecules is essential. Therefore, the carrier transition rate of a host, guest, and SiQDs in a phosphorescent OLED (PhOLED) should be considered. The relationship of transition rate in the guest–host system has been studied previously [33]. We included SiQDs in the conventional guest–host transition system. The transition rate inside a proposed system contains K_H_, K_F_, K_R_, K_G_, K_FF_, K_RR,_ and K_QDs_, as displayed in Fig. 1c. Where K_H_, K_G_, and K_QDs_ are the radiative recombination rate in the host, guest, and the SiQDs, respectively.

The K_F_ and K_R_ are carriers’ forward and reverse transition rates from the host to the guest material. K_FF_ and K_RR_ are carriers’ forward and reversed transition rates from the guest to the SiQDs. K_G_ should be maximized to achieve the highest luminescent efficiency. Therefore, either K_G_ should be more significant than K_H_ or K_F_, or it should be more effective than K_R_ and K_H_ between host and guest materials, as shown in Fig. 1d. Furthermore, K_G_ should either be higher than KQDs or KRR should be more significant than K_FF_ and K_QDs_ to prevent radiative recombination in the SiQDs.

### Silicon quantum dots preparation

To prepare SiQDs, the hydrogen silsesquioxane (HSQ) was used as a precursor and was used as received. The solvent was removed from the HSQ stock solution using a rotary evaporator in a water bath at 40 °C, resulting in gel formation and drying overnight under vacuum. After it dried, the white solid was placed in a quartz crucible and transferred in an inert atmosphere to a high-temperature furnace, where it was annealed at 1100 °C for one hour in the atmosphere of 95% Ar and 5% H_2_. After grinding, 200 mg of the fine powder was added to a mixture of 2 mL of de-ionized (DI) water, 2 mL of ethanol, and 2 mL of HF to etch the SiO_2_ matrix and decrease the size of the Si. After solvent removal and centrifugation, the product was dried under a dry N_2_ flow to obtain 20 mg of powder and quickly transferred to a round-bottom flask containing 10 mL of 1-dodecane. The solution was then heated at 190 °C overnight in Ar ambient in the same round-bottom flask. After the reaction and removal of the excess 1-dodecane, 5 mL of toluene was added to obtain a 1-dodecane-capped, hydrogen-terminated SiQDs solution. The solution is further diluted to 0.1 wt % in toluene for device preparation.

### Quantum dot *characterization*

The TEM images of SiQDs are shown in Fig. 1e, and the SiQDs are well separated. The size of the SiQDs ranged from 3.1 to 9.0 nm and had the highest population at around 6.6–7.0 nm, as shown in Fig. 1f, according to the TEM images. However, the enlarged TEM images (as shown in Fig. 5c) indicate that a few Si QDs exhibit diameters less than 3 nm. The smaller Si QDs may have a larger bandgap [34], and the conduction band edge and the valence band edge may exceed the lowest unoccupied molecule orbit (LUMO) and highest occupied molecule orbit (HOMO) of the luminescent materials that enabling carrier transport, which will be discussed in Sect. 3.4. The photoluminescence of the Si QD is shown in Fig. 1g. It peaks at around 680 nm, a red emission corresponding to the bandgap of 1.82 eV. After blending the SiQDs with a 5 × 10^–3^ wt.% concentration to the host (4,4’-Bis(N-carbazolyl)-1,1’-biphenyl (CBP)) and the guest Bis[2-(4,6-difluorophenyl) pyridinato-C2, N] (picolinato) iridium (III)(FIrpic)) materials in the precursor. Three PL emissions at 436 nm, 470 nm, and 510 nm were found for the blending solution with and without SiQDs, corresponding to the CBP and FIrpic emissions, respectively. The PL emission associated with SiQDs was not observed in this system. Further, excite the solution by 254 nm UV light, the SiQDs exhibit a red color, and FIrpic + CBP and FIrpic + CBP + SiQDs solutions display a sky-blue color, as shown in Fig. 1h, which coincides with the PL spectrum. This indicates the additive of the SiQDs does not alter the emission of the guest and host system in the phosphorous OLED. These observations support our assumption that the additive SiQDs have small KQDs and observable radiative luminescence and are used to provide the carriers to the emitting molecules, the FIrpic.

### SiQDs OLED *device fabrication*

**Fig. 2:**
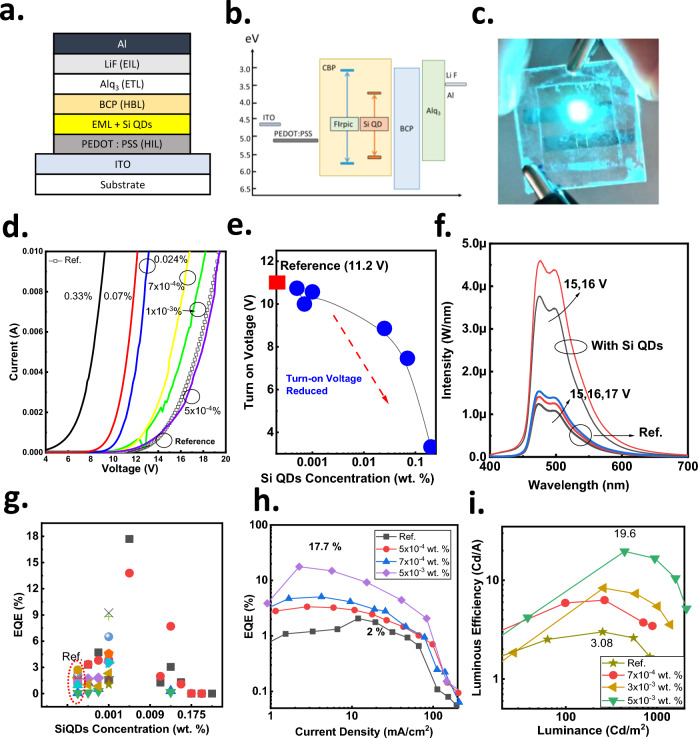
The structure and performance of the SiQDs doped CGCs PhOLED. **a**, **b** The layer structure and energy alignments of the PhOLED. The thickness of PEDOT PSSS, EMI + SiQDs, BCP, Alq_3_, LiF, and Al are 30, 68, 10, 20, 1, and 100 nm, respectively. **c** The photograph of the sky-blue emission from the PhOLED. **d** The current–voltage characteristics of the PhOLED device with different SiQDs concentrations. **e** The dependence of the turn-on voltage on the SiQDs concentration. **f** The emission spectrum of the OLED from the referenced and SiQDs doped (5 × 10^−3^ wt.%) device. **g** The dependence of EQE on the concentration of SiQDs in the precursor. **h** the EQE of the SiQDs doped CGCs devices to the current density, and **i** the luminescent efficiency of the SiQDs doped CGCs devices to the luminance

The layer structure and energy alignments of the PhOLED were depicted in Fig. 2a and b, respectively. Indium-tin-oxide (ITO) coated glass was used as a substrate to make SiQDs OLED devices. The ITO was patterned into two mm-wide slots. After cleaning, organic layers were applied. The conducting polymer, poly(3,4-ethylenedioxythiophene)-poly(styrenesulfonate) (PEDOT: PSS), was used as the hole injection layer, spin-coating on the ITO at 5,000 rpm for the 30 s. After coating, the PEDOT: PSS was thermally dried on a hot plate at 120 °C for 10 min. The thickness is approximately 30 nm. After PEDOT: PSS, the precursor of the emission layer was also spin-coated on the PEDOT: PSS at 2,000 for the 30 s and was then dried on a hotplate at 40 °C for 240 s. FIrpic is dissolved with SiQDs in the CBP as the emitting layer. The 4,4’-Bis(N-carbazolyl)-1,1’-biphenyl (CBP) and Bis[2-(4,6-difluorophenyl) pyridinato-C2, N] (picolinato) iridium (III)(FIrpic) were dissolved in the chlorobenzene (CB) in a total concentration of 2.4 wt. % in 1 ml as precursors. The weight of CBP is 21.12 mg, which is 2.88 mg for FIrpic. All chemicals in the precursors of the emitting layer (EML) were used as received. The SiQDs in the solution were added into the precursors by a micropipette with different amounts. The PEDOT: PSS and light-emitting layer thickness are approximately 30 and 68 nm measured by a stylus profiler (BRUKER DektakXT) and was confirmed by an atomic force microscopy (Digital Instruments Dimension 3100 Controller). After the emitting layer, 2,9-Dimethyl-4,7-diphenyl-1,10-phenanthroline (BCP), Tris(8-hydroxyquinolinolato)-aluminum (III)(Alq3), Lithium Fluoride (LiF), Aluminum (Al) were sequentially coated in 10, 20, 1 and 100 nm thick, respectively, by thermal evaporation. The device area is 2 mm by 3 mm.

## Results and discussion

### Characterization of OLED emission

The photograph of the sky-blue emission from the PhOLED is shown in Fig. 2c. The current–voltage characteristics of the PhOLED device with different SiQDs concentrations are shown in Fig. 2d**,** which was measured using an Agilent B2912A semiconductor parameter analyzer in dark conditions. The PhOLED were made of the SiQDs in the precursor from 5 × 10^–4^ to 0.33 wt.%, a significant range of doping concentration. With a high SiQDs concentration (0.33 wt. %), the turn-on voltage is around 3.3 V, which is a 7.9 V reduction compared to the reference device.

This indicates the injected carriers may transport through the SiQDs, causing a concentration quench. Therefore, no emissions are recorded until the SiQD concentration is reduced to 0.11 wt.%. In contrast to OLED with layer CGLs, the PhOLED with SiQDs CGCs has reduced operation voltage instead of increased. The conventional peripheral circuit can operate the SiQDs CGCs PhOLED in low voltage. The dependence of the turn-on voltage on the SiQDs concentration is plotted in Fig. 2e. The turn-on voltage increased with decreased SiQDs concentration in the precursors. With 5 × 10^–4^ wt. % of the SiQDs, the turn-on voltage is almost identical to the reference device, indicating that the injected carriers will not transport through SiQDs. The emission spectrum of the OLED from the reference and SiQDs doped (5 × 10^–3^ wt. %) device was shown in Fig. 2f taken by a spectroradiometer (Optronic Laboratories, OL-770) equipped with a 6-inch integrated sphere powered by a Keithley 2400 source meter. The applied voltage is indicated in the figure. They have almost identical peaks in the emission, 470 and 510 nm. However, PhOLED with SiQDs CGCs exhibits lower turn-on voltage and higher external quantum efficiency (EQE). The EQE increased with the increase of the SiQDs concentration from 5 × 10^–4^ to 5 × 10^–3^ wt. %. Meanwhile, the SiQDs concentration is beyond 5 × 10^–3^ wt. %, the EQE starts to decrease, as shown in Fig. 2g. The highest EQE for the PhOLED with SiQDs is 17.7% at low current density (2 mA/cm^2^), as shown in Fig. 2h. In comparison, the EQE for the PhOLED without SiQDs is only 2% in this study. The luminous efficiency for the device is 19.6 cd/A at 500 cd/m^2^ for the PhOLED with SiQDs, as shown in Fig. 2i. In comparison, it is 3.08 cd/m^2^ for the reference device at a much lower luminance. The EQE enhanced about eight times from 2 to 17.7% for the SiQDs doping.

### Distribution of SiQDs

**Fig. 3:**
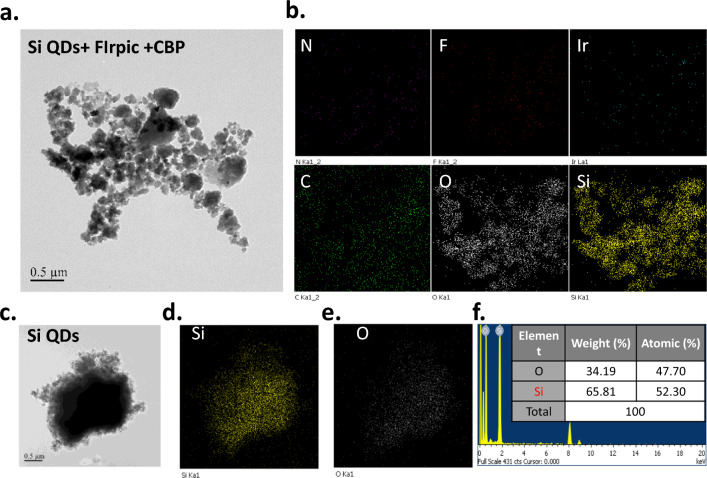
The TEM images of SiQDs and luminescent precursors containing CBP, FIrpic, and SiQDs. **a** The TEM images of the luminescent precursor containing CBP, FIrpic, and SiQDs. **b** The areal distribution of element atoms (N, F, Ir, C, O, and Si). **c** The TEM images of the SiQDs. **d**, **e** The distribution of Si and O atoms in the SiQDs. **f** The atomic percentage of Si and O in the SiQDs obtained from EDS

The additive of quantum dots or nano-flasks into the precursor solution of the luminescent materials may be thought to condense at the hole injection layer (HIL) and emitting layer (EML) [35], which enhances the hole injection efficiency and hence improves the EQE. To clarify that the SiQDs are well-dispersed inside the emitting materials instead of forming a layer at the HIL/EML interface, the areal and vertical distribution of the SiQDs in the EML were explored. The precursor solution contains CBP, FIrpic, and 5 × transmission electron microscopy TEM investigated 10^–3^ wt. % of the SiQDs). As shown in Fig. 3a, the dropped solution on the Cu mesh tends to aggregate together. The elements analysis indicates the Si atoms have a distribution shape the same as the TEM image, as shown in Fig. 3b. It is worth noting that the O atoms have the same distribution as the Si atoms. Although the N, F, Ir, and C atoms cannot be observed to have the same distribution shape as the TEM image, they are distributed well. These atoms are associated with the CBP and FIrpic. The TEM images indicate the SiQDs are well dispersed in the CBP and FIrpic blended materials. Further exploring the SiQD, the shape of TEM images (Fig. 3c), the distribution of the Si atoms (Fig. 3d), and the oxygen atoms (Fig. 3e) are identical, which indicates the SiQDs contain large amounts of oxygen. The atomic percentage of oxygen is like the Si atoms (Fig. 3f).

**Fig. 4:**
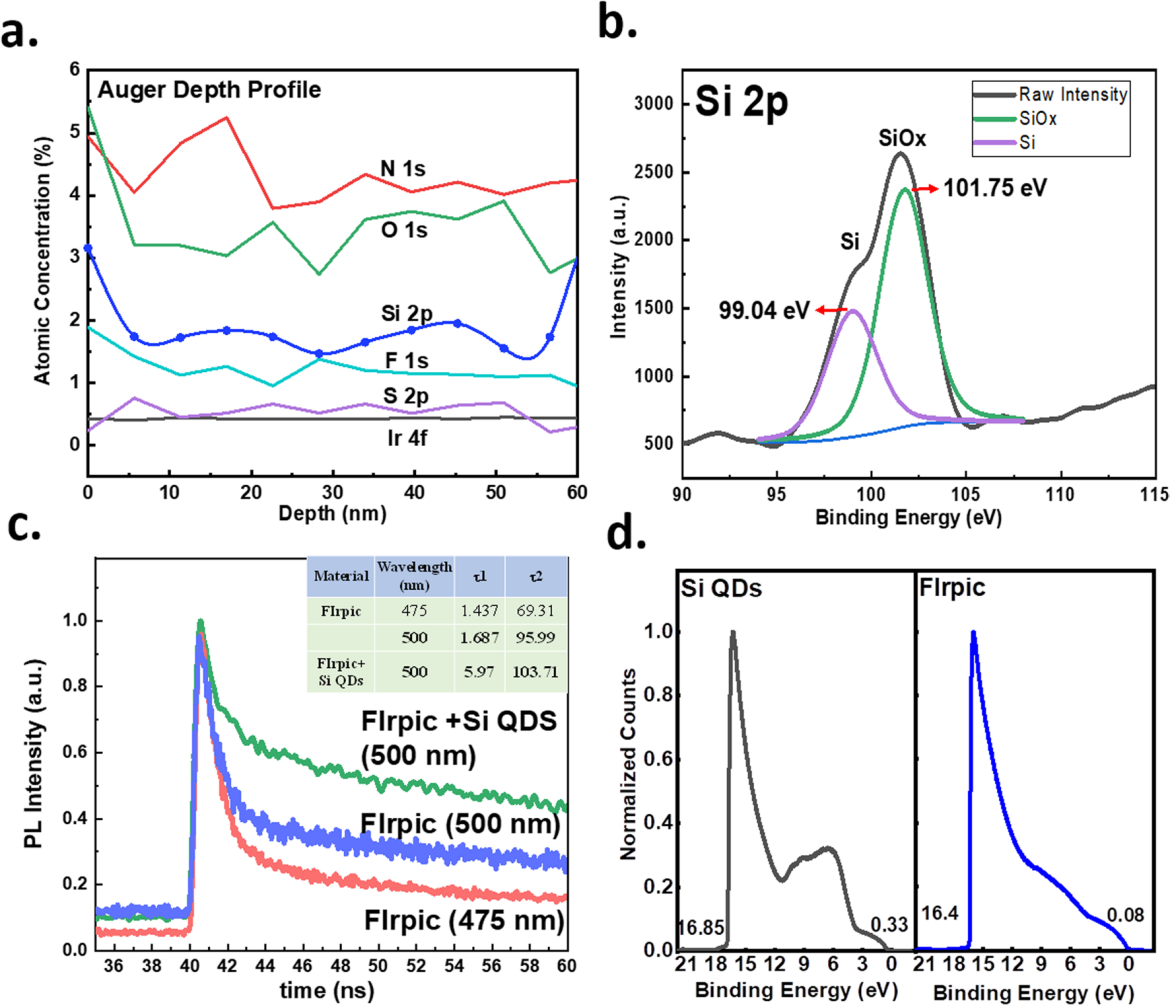
The spectroscopy analysis of the SiQDs CGCs luminescent materials. **a** The depth profile of N 1s, O 1s, Si 2p, F 1s, Ir 4f, and S 2p signals studied by XPS. **b** The binding energy spectrum of Si 2p signal. It was deconvoluted into two distributions corresponding to the Si–Si and Si–O bonds. **c** The TR-PL spectrum of the Firpic and Firpic blended with SiQDs at 500 nm wavelength. **d** The UPS spectrum of SiQDs and the Firpic with the calibration of Au signal

The SiQDs doped (5 × 10^–3^ wt. %) Firpic and CBP thin film was investigated by the x-ray photoemission (XPS) depth profile to understand the vertical distribution. The depth is deduced from the XPS’s sputter time and film thickness. The atomic signals of Si and O exhibit stable distribution from the top of the emitting layer to the bottom, as shown in Fig. 4a. The signals of F and Ir were also analyzed. They are indicators of the FIrpic. Si, O, F, and Ir coexistence indicates the SiQDs are distributed well along the film thickness with the Firpic. The TEM and XPS studies show that the SiQDs are well-dispersed spatially inside the EML and do not pile up at the HTL/EML interface. In addition to the SiQDs distribution, the binding status of the Si is studied by XPS, as shown in Fig. 4b. The Si 2p spectroscopy can be deconvoluted into two peaks at 99.6 eV and 102.36 eV, which corresponds to the Si and SiOx. The existence of the SiOx indicates the SiQDs may partially oxidize at its outer surface, forming a SiOx/Si core–shell structure that prevents the coalescence of SiQDs.

### Carrier dynamics in the OLED

The donating of carriers from SiQDs to the FIrpic can be proved by the time-resolved PL (TRPL). The transient behavior in the TRPL can be modeled by the exponential decay with a time constant [36]. The time constant represents the lifetime of carriers in the material system that could contribute to the radiative light emission. The TRPL from the FIrpic + CBPP and SiQDs doped FIrpic + CBPP are depicted in Fig. 4c. The TRPL presents two distinct decay behavior. Therefore, it is modeled by the following equation with two lifetimes.

1 $$I_{PL} = A_{1} e^{{ - \left( {{\raise0.7ex\hbox{${t - t_{0} }$} \!\mathord{\left/ {\vphantom {{t - t_{0} } {\tau_{1} }}}\right.\kern-0pt} \!\lower0.7ex\hbox{${\tau_{1} }$}}} \right)}} + A_{2} e^{{ - \left( {{\raise0.7ex\hbox{${t - t_{0} }$} \!\mathord{\left/ {\vphantom {{t - t_{0} } {\tau_{2} }}}\right.\kern-0pt} \!\lower0.7ex\hbox{${\tau_{2} }$}}} \right)}}$$

The τ_1_ and τ_2_ for referenced and SiQDs doped devices at 510 nm are fitted by this equation. The τ_1_ and τ_2_ are 1.68 and 95.99 ns for the referenced device and 5.97 and 103.71 ns for the device with SiQDs doping. The τ_1_ is 3.5 times enhanced after SiQDs doping. The lifetime can be further correlated with the transition rate by the following equation,

2 $${\tau }_{PL}=\frac{1}{{K}_{r}+{K}_{nr}}$$

in which the K_r_ is the transition rate of radiative recombination, and the K_nr_ is the transition rate of non-radiative recombination. According to Fig. 1c and d, the difference in transition rate between reference and SiQDs-doped material systems is the forward and backward carrier transition rates, K_FF_ and K_RR_. Therefore, the ${\tau }_{PL}$ in the reference can be expressed as

3 $${\tau }_{PL}=\frac{1}{{K}_{G}+{K}_{nr}}$$

The ${\tau }_{PL, Si QDs}$ for the materials containing SiQDs can be expressed as

4 $${\tau }_{PL, Si QDs}=\frac{1}{{K}_{G}+{K}_{nr}+{K}_{FF}-{K}_{RR}}$$

If K_RR_ is larger than K_FF_, the denominator in the (4) will be smaller than in the (3). Consequently, the ${\tau }_{PL, Si QDs}$ is larger than ${\tau }_{PL}$, which coincides with the TRPL results in this work. The more extensive lifetime in the SiQDs doped layer indicates the carriers in the SiQDs are donating to the FIrpic, as our assumption.

### Carrier transport path in the OLED

**Fig. 5:**
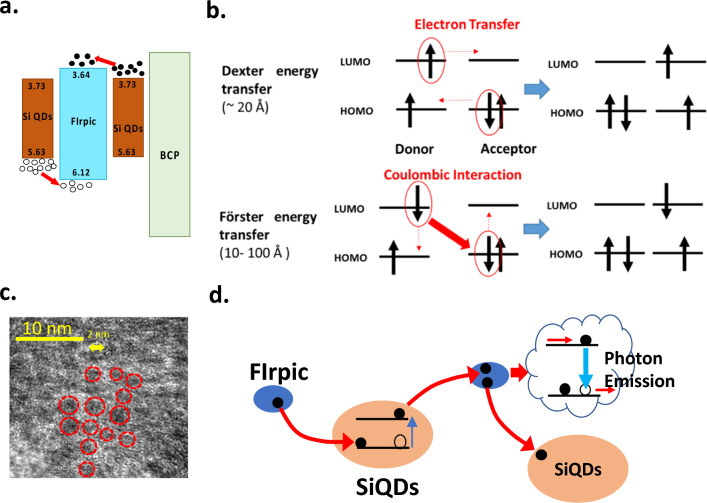
The mechanism of charge transfer in the SiQDs CGCs system. **a** The energy alignments between SiQDs and FIrpic according to the UPS measurement. **b** The schematic expression of the Dexter and Föster energy transfer. The Föster transfer is a Coulombic resonant energy transfer, and the Dexter transfer corresponds to electron transfer. **c** The SiQDs (marked by the red circle) are identified in the TEM images, with the distance between them being 2 nm on average. **d** The schematic diagram shows how carriers in the SiQDs are transferred to Firpic through Dexter electron transfer

The ultra-violet photoemission spectroscopy (UPS) is used to study the actual band alignment between SiQDs and the Firpic. The UPS for SiQDs and Firpic are shown in Fig. 4d. The energy of the input UV photons is 21.2 eV, and the Fermi levels extracted for SiQDs and Firpic are 4.68 eV and 4.88 eV, respectively. We assume either SiQDs or Firpic are undoped, and the measured Fermi levels are the mid-gap. The conduction band and valence band edges of the SiQDs are calculated as 3.73 and 5.63 eV, respectively. With the same method, the calculated LUMO and HOMO of FIrpic are 3.64 and 6.12 eV, respectively. The alignment between SiQDs and the FIrpic is plotted in Fig. 5a. The barrier for electron transfer from SiQDs to the FIrpic is 0.09 eV, which is easily. And the energy barrier is 0.49 eV for the holes in the SiQDs. The average thermal energy of the carrier is 3/2 kT, which is around 0.04 eV at room temperature. These barriers are higher than the thermal energy, and the holes in the SiQDs in the valence band edge seem not to overcome this barrier easily. The HOMO of the FIrpic is also reported to be 5.8 eV, which is 0.32 eV smaller than we obtained in this work [37].

Please take note of the following information:

The reduced energy level of the HOMO in FIrpic indicates that the energy barrier for holes in Si QDs to transfer is reduced to 0.17 eV. This reduced barrier implies that the transfer probability is increased. Additionally, the valence band edge of Si QDs is also subject to change. As observed in Fig. 1f, the diameter of Si QDs ranges from 3 to 9 nm. Furthermore, in the enlarged TEM images, some Si QDs with diameters smaller than 2 nm were also observed (Fig. 5c). The bandgap of the Si QDs is proved to increase as the diameter decreased [34]. For example, Si QDs with a diameter of 1.9 nm have a bandgap of 2.2 eV, while those with a 3 nm diameter exhibit a bandgap of approximately 2 eV [38]. These bandgaps are higher than the bandgap of Si QDs calculated from the PL emission (Fig. 1g), which is 1.82 eV. Considering the PL emission covering from 580 to 760 nm, the bandgap of Si QDs ranges from 1.63 to 2.14 eV. Therefore, some Si QDs exhibit a much lower energy barrier for the holes to transfer to FIrpic.

The reason for there is an appropriate concentration of the SiQDs doping can be understood by the mechanism of carrier transfer from SiQDs to the surrounding emitting molecules, FIrpic. With high concentrations of the SiQDs, large amounts of charges are transported through SiQDs, proved by the increased current density as explored. As we demonstrated, the transport between SiQDs causes a concentration quenching because the SiQDs have a low radiative recombination rate. Reducing the SiQDs’ concentration can help reduce the transport path between SiQDs. Therefore, the isolated charges inside SiQDs can donate to the neighbor luminescent molecules, the FIrpic.

The EQE reaches a maximum when the transport path through SiQDs is prohibited, and most of the luminescent molecules are ranged in the donation range of the SiQDs. Two mechanisms are responsible for the charge transfer in the organic system, as shown in Fig. 5b: Dexter charge transfer and Föster resonant energy transfer (FRET) [39, 40].

The electron in the SiQDs may transfer directly to the closed-surrounded luminesce molecules by the Dexter charge transfer, setting up the charge donation [40]. By the FRET, the energy that recombines an electron in the SiQDs from the conduction band to the valence transfers to the other luminescent molecule by exciting an electron from the ground to the excited state [40]. We marked part of the SiQDs in the TEM image, as shown in Fig. 5c, to verify a possible transfer mechanism. Each SiQD is very close, within 2 nm, for the doping level of SiQDs is 5 × 10^–3^ wt. %. Therefore, the dominant carrier transfer mechanism is most possibly the Dexter direct transfer. The electrons in the SiQDs are transferred directly to luminescent molecules, as schematically shown in Fig. 5d. The thermally generated electron at the conduction band of the Si QDs is transferred to the excited state of the nearest FIrpic. Meanwhile, the electron in the ground state of the FIrpic is transferred to the valence band of the Si QDs to maintain the charge neutrality. The electron in the excited state may transition to the empty ground state radiatively by emitting a photon. These internal charge transfer and radiative recombination processes enhance the external quantum efficiency. External quantum efficiency is improved through the transfer of carriers from the quantum dots to the luminescent materials, which depends on the energy barrier between them. This improvement may be applicable to other material systems with similar energy barriers. Quantum dots with appropriate band alignment might enhance the EQE in the OLED with green or red emission organic luminescent materials.

## Conclusion

Silicon quantum dots are immersed in this work into phosphorescent luminescent materials as charge generation centers for unlimited electrons and holes. The SiQDs have an average diameter of 6 nm and are well dispersed in the luminescent materials by TEM and XPS analysis. The PhOLED exhibits 17.7% external QE by the SiQDs doping in minimal amounts of 5 × 10^–3^%, which is approximately 9 times enhancement. TRPL measured the decay time of radiative emission on the luminescent organic with SiQDs, and the increased decay time at the luminescent wavelength (500 nm) reveals the supply of excitons from the neighbors, which are SiQDs. The proposed and demonstrated concept solely involved electron transfer that may not limited by the categories of inorganic quantum dots, the emission wavelength, and the selection of luminescent organics.

## Data Availability

The data that support the plots and other findings within this report are available from the corresponding authors upon reasonable request.

## Abbreviations

EQE: External quantum efficiency
OLED: Organic light-emitting diodes
PhOLED: Phosphorescent OLED
SiQDs: Silicon quantum dots
CGCs: Charge-generation centers
EML: Emitting layer
Firpic: Bis[2-(4,6-difluorophenyl) pyridinato-C2, N] (picolinato) iridium (III)
CBP: (4,4’-Bis(N-carbazolyl)-1,1’-biphenyl
PEDOT:PSS: Poly(3,4-ethylenedioxythiophene)-poly(styrenesulfonate)
TRPL: Time-resolved photoluminescence
UPS: Ultraviolet photoelectron spectroscopy
LUMO: Lowest unoccupied molecule orbit
HOMO: Highest occupied molecule orbit
FRET: Föster resonant energy transfer
